# Comment on: Radiofrequency Versus Myofascial Therapy in Women with Myofascial Chronic Pelvic Pain: A Randomized Clinical Trial

**DOI:** 10.1007/s00192-026-06755-9

**Published:** 2026-07-08

**Authors:** Markus Huebner

**Affiliations:** 1 Department of Obstetrics and Gynecology, Medical Center, University of Freiburg, Hugstetter Str. 55, 79106 Freiburg, Germany; 2 Faculty of Medicine, University of Freiburg, Freiburg, Germany

The article “Radiofrequency versus myofascial therapy in women with myofascial chronic pelvic pain: a randomized clinical trial” by Box et al. [1] analyzed the efficacy of an isolated radiofrequency therapy in patients with chronic pelvic pain. This condition affects up to 26.6% of women and significantly decreases health-related quality of life [2]. Urogenital and overall functionality can be impaired [3]. There is standard conservative treatment such as myofascial manual therapy [4]; however, it remains unclear, whether or not monopolar radiofrequency has any benefits when used alone.

The authors set up a three-arm randomized controlled trial in an university hospital setting. Active radiofrequency therapy was compared with sham radiofrequency and myofascial manual therapy. A fourth group represented an observational cohort. The administration of radiofrequency therapy was double blinded. Outcome parameters were pain intensity, quality of life, and sexual function. A total of 80 women were randomized into four groups with 20 women within each group. The use of radiofrequency therapy significantly reduced pain in women with chronic pelvic pain (mean decrease 3.98 points on the NRS, 95% CI 2.85–5.10; *p* < 0.001). Quality of life increased in both radiofrequency groups. Regarding sexual function, no differences could be found.

Patients with chronic pelvic pain suffer in a special way, with a significant impact on their quality of life. Box et al. answered the questions regarding radiofrequency therapy in these patients in a double-blinded, prospective randomized controlled trial, the best way to test research hypotheses. I would like to congratulate the authors on their work.
